# Tryptophan Photoproduct FICZ Upregulates IL1A, IL1B, and IL6 Expression via Oxidative Stress in Keratinocytes

**DOI:** 10.1155/2018/9298052

**Published:** 2018-11-25

**Authors:** Yuka Tanaka, Hiroshi Uchi, Akiko Hashimoto-Hachiya, Masutaka Furue

**Affiliations:** ^1^Department of Dermatology, Graduate School of Medical Sciences, Kyushu University, Maidashi 3-1-1, Higashi-ku, Fukuoka 812-8582, Japan; ^2^Research and Clinical Center for Yusho and Dioxin, Kyushu University Hospital, Maidashi 3-1-1, Higashi-ku, Fukuoka 812-8582, Japan; ^3^Division of Skin Surface Sensing, Department of Dermatology, Graduate School of Medical Sciences, Kyushu University, Maidashi 3-1-1, Higashi-ku, Fukuoka 812-8582, Japan

## Abstract

Ultraviolet B (UVB) irradiation activates the aryl hydrocarbon receptor (AHR), generates the reactive oxygen species (ROS), and induces the production of proinflammatory cytokines such as IL1A, IL1B, and IL6. 6-Formylindolo[3,2-b]carbazole (FICZ) is a tryptophan-derived photoproduct that is induced by UVB irradiation and activates the AHR. However, its role in upregulating proinflammatory cytokine expression has never been investigated. Here, we demonstrated that FICZ enhanced ROS generation in human HaCaT keratinocytes in an AHR-dependent manner. FICZ also upregulated the expression of *IL1A* and *IL1B*, as well as the expression of *IL6* and the production of its protein product, in an AHR- and ROS-dependent fashion. Here, we demonstrate that the actions of FICZ can substitute for the hazardous effects of UVB exposure, contributing to the further understandings of the mechanisms which UVB harms organisms.

## 1. Introduction

Skin is a specialized sense organ for external stimuli such as ultraviolet (UV) irradiation and environmental pollutants. UV exposure accelerates photoaging and photocarcinogenesis by generating reactive oxygen species (ROS) and proinflammatory cytokines such as IL1A, IL1B, and IL6 [1–5]. Previous studies have shown that dioxins and UVB share, at least in part, a signal transduction pathway via the aryl hydrocarbon receptor (AHR) [6, 7]. Activation of the AHR by dioxins upregulates the transcription of responsive genes such as *cytochrome P450 1A1* (*CYP1A1*) and induces the production of ROS and proinflammatory cytokines in keratinocytes [7–9]. Notably, UV radiation upregulates the AHR-CYP1A1 axis in rat and human skin *in vivo* [10, 11] and in human HaCaT keratinocytes *in vitro* [12].

Fritsche's, Rannug's, and Krutmann's groups searched for photoproducts that are capable of activating the AHR-CYP1A1 system and found that the tryptophan-derived photoproduct 6-formylindolo[3,2-b]carbazole (FICZ) is generated under the exposure to UV radiation and it possessed the ability to activate AHR-CYP1A1 system [6, 13–15]. UVB induces conformational changes in intra- and extracellular tryptophan and generates FICZ [6]. FICZ is a high-affinity ligand for AHR and upregulates CYP1A1 production [6, 16–18]. FICZ also contributes to the increased or decreased production of cyto/chemokines including IL6 and CCL5 [19, 20]. Therefore, the actions of FICZ can be substituted for at least part of UVB-mediated biological activities, which adversely affect organisms. However, it remains unknown whether the FICZ-AHR-ROS pathway upregulates IL1A, IL1B, and IL6 expression in keratinocytes.

We demonstrated here that FICZ (1) activated the AHR-ROS pathway and (2) upregulated IL1A, IL1B, and IL6 expression in an AHR- and ROS-dependent manner.

## 2. Materials and Methods

### 2.1. Reagents

FICZ (Enzo Life Sciences, Farmingdale, NY) was dissolved in dimethyl sulfoxide (DMSO; Sigma-Aldrich, St. Louis, MO) and added to Dulbecco's modified Eagle's medium (DMEM; Sigma-Aldrich) at final concentrations of 1, 10, 100, and 1000 nM. Benzo[a]pyrene (BaP; Sigma-Aldrich) and N-acetyl-L-cysteine (NAC; Sigma-Aldrich) were dissolved in DMSO and added to the culture medium at final concentrations of 1 and 5 *μ*M, respectively. An oxidation-sensitive dye, carboxy-H_2_DCFDA (Thermo Fisher Scientific, Waltham, MA), was dissolved in DMSO at a concentration of 10 mM and further diluted in HBSS (Fujifilm Wako Pure Chemical Corporation, Osaka, Japan) at a final concentration of 25 *μ*M. CH223191 (Merck, Darmstadt, Germany), an AHR antagonist, was dissolved in DMSO and added to culture medium at a final concentration of 10 *μ*M. The antibodies used were rabbit anti-human *β*-actin antibody (Cell Signaling Technology, Danvers, MA), rabbit anti-human AHR antibody (H-211) (Santa Cruz Biotechnology, Dallas, TX), rabbit anti-human NF-*κ*B p65 (Abcam, Cambridge, United Kingdom), and horseradish-peroxidase-conjugated anti-rabbit secondary antibody (Cell Signaling Technology).

### 2.2. Cell Culture

HaCaT cells—an immortalized human keratinocyte cell line—were maintained in DMEM supplemented with 10% fetal bovine serum (FBS), 100 units/mL penicillin, and 100 *μ*g/mL streptomycin (Thermo Fisher Scientific). Cells were passaged every 2 to 3 days. Normal human epidermal keratinocyte (NHEK) was maintained in KGM-Gold medium (Lonza, Basel, Switzerland) as manufacturer's instruction and passaged at 70-80% confluent.

### 2.3. Cell Viability

The viability of HaCaT cells was determined by using a Cell Counting Kit-8 (Dojindo Molecular Technologies, Inc., Kumamoto, Japan) in accordance with the manufacturer's instructions.

### 2.4. Flow Cytometry

Cells were seeded at a density of 1.2 × 10^5^ cells/well of a 12-well culture plate and incubated for 48 h at 37°C. Cells were then treated with DMSO (control), FICZ (1, 10, 100, or 1000 nM), or BaP (1 *μ*M) in the presence or absence of NAC (5 mM) or CH223191 (10 *μ*M) for 6 h at 37°C. After being washed with Dulbecco's phosphate-buffered saline (DPBS), cells were incubated with carboxy-H_2_DCFDA (25 *μ*M) in HBSS for 30 min at 37°C in the dark and then harvested by trypsinization. Harvested cells were suspended in DPBS containing 5% BSA, 2 mM EDTA, and 0.1% propidium iodide (PI; Thermo Fisher Scientific), and the intensity of dichlorofluorescein (DCF) was measured with a FACSCanto II flow cytometer (BD Biosciences, Franklin Lakes, NJ). The mean fluorescence intensity of DCF was analyzed with FlowJo software (Tree Star, Inc., San Carlos, CA).

### 2.5. Glutathione (GSH) Reduction Assay

HaCaT cells were treated with DMSO (0.1%, control) or FICZ (1, 10, 100, or 1000 nM) for 6 h and analyzed by GSH reduction assay using GSSG/GSH Quantification Kit (Dojindo Molecular Technologies, Inc.) according to the manufacturer's instructions. Briefly, cells were lysed with 10 mM hydrochloric acid (Sigma-Aldrich) and 2 cycles of freeze-thaw process. The lysate was mixed with 5% sulfosalicylic acid (Fujifilm Wako Pure Chemical Corporation), diluted with distilled water, and used for the analysis. The reaction products were quantified with an iMark micro plate reader (Bio-Rad, Hercules, CA) by measuring the absorbance at 415 nm. The amount of GSH was calculated by subtracting the amount of glutathione disulfide (GSSG) from the amount of whole GSH.

### 2.6. siRNA Transfection

Cells were seeded at a density of 1.2 × 10^5^ cells/well of a 12-well culture plate and transfected with Negative Control#1 siRNA or AHR siRNA (s1200; both were purchased from Applied Biosystems, Foster City, CA) by using HiPerFect Transfection Reagent (Qiagen, Hilden, Germany) in accordance with the manufacturer's instructions. After 48 h, the cells were treated with DMSO (0.1%, control), BaP, or FICZ for 6 h, and the DCF intensity was analyzed by flow cytometry as mentioned above.

### 2.7. Quantitative Reverse Transcription–Polymerase Chain Reaction (qRT-PCR)

Total RNA was extracted from cells by using an RNeasy Mini Kit (Qiagen) and reverse transcribed by using a PrimeScript RT reagent Kit (TaKaRa Bio Inc., Shiga, Japan). PCR was performed with TB Green Premix Ex Taq II (TaKaRa Bio Inc.) in accordance with the manufacturer's instructions. The qRT-PCR amplification cycles were 95°C for 30 s, followed by 40 cycles of 95°C for 5 s, and 60°C for 20 s. Expression levels of each target gene were normalized against the cycle threshold of the *β*-actin gene (*ACTB*). The sequences of the primers used are listed in Table 1.

### 2.8. Enzyme-Linked Immunosorbent Assay (ELISA)

HaCaT cells were seeded at a density of 3.0 × 10^5^ cells/well of a six-well culture plate and incubated for 48 h at 37°C. Cells were then treated for 6 or 12 h with DMSO (0.1%, control) or FICZ (100 nM) in the presence or absence of NAC (5 mM). After the incubation, culture supernatant was collected, and the concentrations of IL-1A, IL-1B and IL-6 were measured by using a Quantikine Human IL-1*α*, IL-1*β*, or IL-6 ELISA Kit (R&D Systems, Minneapolis, MN) in accordance with the manufacturer's instructions. Absorbance was measured with an iMark microplate reader (Bio-Rad, Hercules, CA).

### 2.9. Western Blotting

Proteins were extracted from siRNA-transfected cells and used for western blotting. Briefly, HaCaT cells were seeded onto six-well plates, and siRNAs were transfected as mentioned above. Forty-eight hours posttransfection, cells were lysed with lysis buffer (25 mM HEPES, 10 mM Na_4_P_2_O_7_·10H_2_O, 100 mM NaF, 5 mM EDTA, 2 mM Na_3_VO_4_, and 1% Triton X-100) and used for SDS-PAGE with 5% to 20% polyacrylamide gel (Bio Craft, Tokyo, Japan). Proteins were then transferred to polyvinylidene difluoride membrane (Merck) and probed with rabbit anti-human *β*-actin antibody or rabbit anti-human AHR antibody. After labeling with horseradish-peroxidase-conjugated anti-rabbit secondary antibody, immunological bands were detected with SuperSignal West Pico Chemiluminescence substrate (Pierce, Rockford, IL) and a ChemiDoc XRS Plus system (Bio-Rad).

For the analysis of NF-*κ*B activation, nuclear and cytoplasmic proteins were separately extracted from the cells using NE-PER Nuclear and Cytoplasmic Extraction Reagents (Thermo Fisher Scientific). Expressions of NF-*κ*B p65 and Lamin B1, an internal control, in nuclear extract were evaluated.

### 2.10. Statistics

Results are presented as means ± standard deviation (SD). The significance of differences between groups was assessed by using Student's unpaired two-tailed *t*-test (two groups) or one-way ANOVA, followed by Tukey's multiple comparisons test (multiple groups) using GraphPad PRISM software (GraphPad Software, La Jolla, CA). A *P* value less than 0.05 was considered statistically significant.

## 3. Results

### 3.1. FICZ Induces ROS Generation in an AHR-Dependent Manner

**Figure 1:**
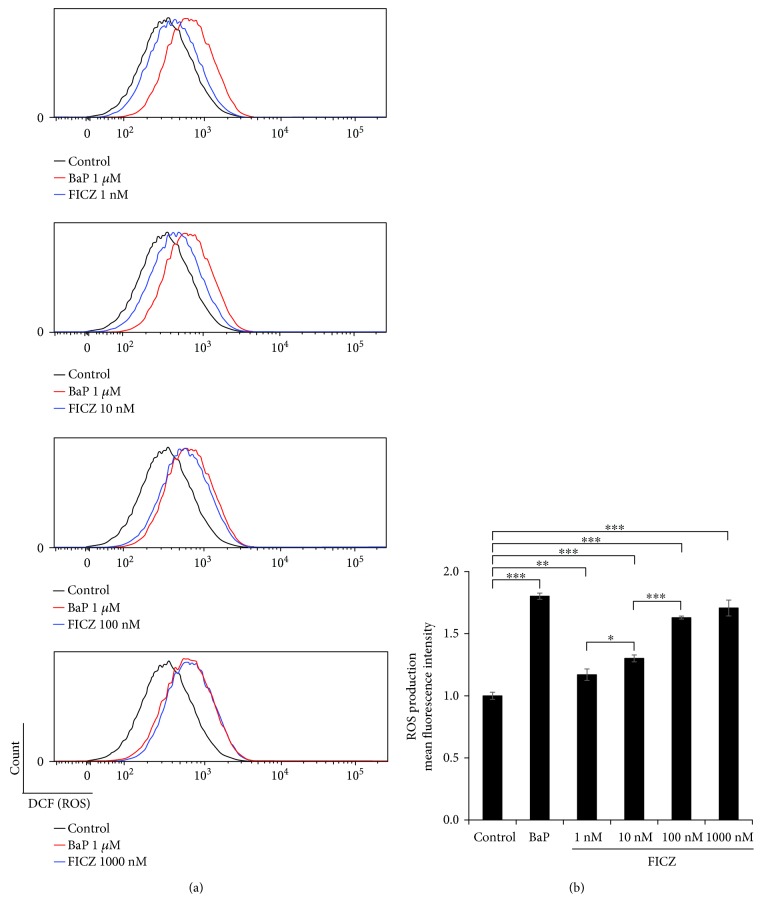
FICZ induces ROS production in a dose-dependent manner. HaCaT cells were treated with DMSO (0.1%, control) or FICZ (1, 10, 100, or 1000 nM) for 6 h, and ROS production was assessed by flow cytometry. Representative histograms of DCF fluorescence (a) are shown, along with the mean fluorescence intensities of DCF (b). Data are presented as means ± standard deviation (*n* = 3 per group). ^∗^ *P* < 0.05, ^∗∗^ *P* < 0.01, ^∗∗∗^ *P* < 0.001.

The viability of HaCaT keratinocytes was assessed by using a WST-8 formazan-based method, and it was not affected by FICZ within the concentrations as high as 10 *μ*M (Supplementary Figure S1). Using flow cytometry, we examined whether ROS were generated by graded concentrations of FICZ (1, 10, 100, and 1000 nM). The potent AHR agonist BaP was used as a positive control. Significant increases in ROS generation were observed compared with the control, even at 1 nM of FICZ (1.17 ± 0.046-fold increase compared with control), and it increased in a dose-dependent manner up to 100 nM (1.63 ± 0.012-fold increase compared with control) in HaCaT keratinocytes (Figure 1).

**Figure 2:**
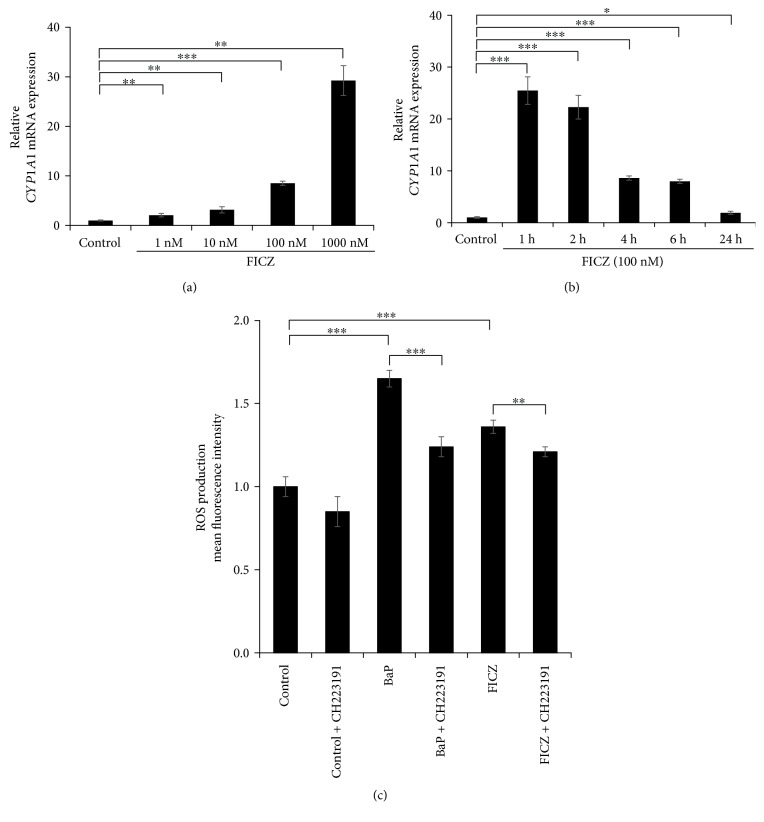
AHR antagonist CH223191 inhibits ROS production by FICZ. (a) HaCaT cells were treated with DMSO (0.1%, control) or FICZ (1, 10, 100, and 1000 nM) for 6 h or (b) were treated with DMSO (0.1%, control) or FICZ (100 nM) for 1, 2, 4, 6, and 24 h, and the mRNA expression of CYP1A1 was assessed by qRT-PCR. (c) HaCaT cells were treated with DMSO (0.1%, control), BaP (1 *μ*M), or FICZ (100 nM) alone or simultaneously with the AHR antagonist CH223191 (10 *μ*M) for 6 h, and ROS production was assessed by flow cytometry. Mean fluorescence intensities of DCF are shown. Data are presented as means ± standard deviation (*n* = 3 per group). ^∗∗^ *P* < 0.01, ^∗∗∗^ *P* < 0.001.

**Figure 3:**
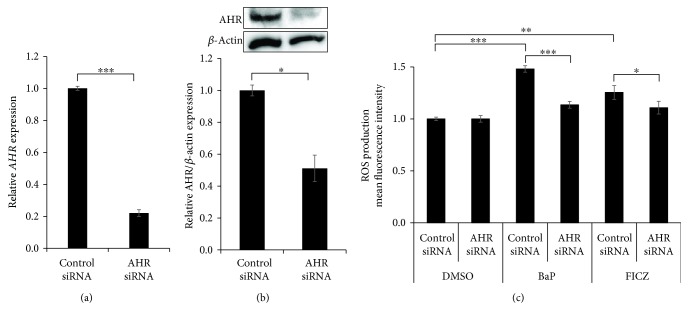
Knockdown of AHR by siRNA inhibits ROS production by FICZ. Negative control siRNA or AHR siRNA was transfected into HaCaT cells, and the efficiency of knockdown of (a) *AHR* mRNA and (b) AHR protein was determined by qRT-PCR and western blotting, respectively. (c) Cells transfected with siRNA were further treated with DMSO (0.1%, control), BaP (1 *μ*M), or FICZ (100 nM) for 6 h. The effects of AHR knockdown on ROS production were assessed by flow cytometry. Data are presented as means ± standard deviation (*n* = 3 per group). ^∗^ *P* < 0.05, ^∗∗^ *P* < 0.01, ^∗∗∗^ *P* < 0.001.

In line with the ROS production, the *CYP1A1* expression was significantly upregulated by 1 to 1000 nM of FICZ (Figure 2(a)) compared to that of DMSO-treated control. Cytoplasmic to nuclear translocation of AHR was also observed even at 1 nM of FICZ (Supplementary Figure S2). In addition, FICZ (100 nM) upregulated the *CYP1A1* expression as early as 1 h after FICZ treatment (Figure 2(b)). To investigate the AHR dependency of FICZ-induced ROS generation, we treated HaCaT keratinocytes with FICZ and simultaneously with the specific AHR inhibitor CH223191 or with AHR siRNA. In the presence of CH223191, BaP-induced or FICZ-induced ROS production was significantly reduced in keratinocytes compared with in the absence of CH223191 (Figure 2(c)). Transfection with AHR siRNA successfully decreased *AHR* mRNA expression (knockdown efficiency; 77.9% ± 0.021%) (Figure 3(a)) and AHR protein production (knockdown efficiency; 48.9% ± 8.28%) (Figure 3(b)). Both BaP-induced and FICZ-induced ROS production were significantly downregulated in AHR-knockdown keratinocytes compared with in control siRNA-transfected keratinocytes (Figure 3(c)). These results indicated that FICZ generated ROS in an AHR-dependent fashion.

In order to neglect the autofluorescence of FICZ [21, 22], we also measured the ROS production by a nonfluorescence-based glutathione reduction assay [23]. FICZ (10 to 1000 nM) did reduce the intracellular level of glutathione, implicating the production of ROS (Supplementary Figure S3).

### 3.2. FICZ Induces IL1A, IL1B, and IL6 Expression in a ROS-Dependent Fashion

**Figure 4:**
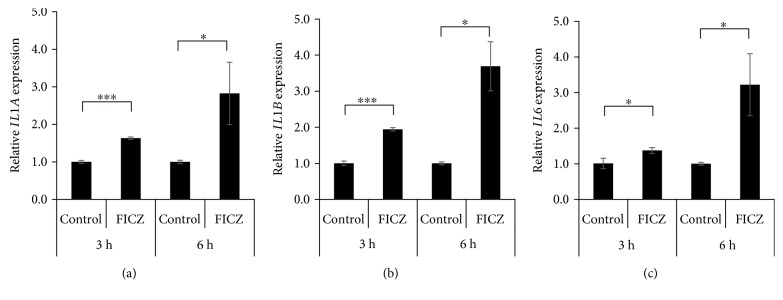
FICZ induces the expression of mRNAs of proinflammatory cytokines. HaCaT cells were treated with DMSO (0.1%, control) or FICZ (100 nM) for 3 or 6 h, and the expression of (a) *IL1A*, (b) *IL1B*, and (c) *IL6* was measured by qRT-PCR. *ACTB* served as an internal control, and relative expression levels compared with those of DMSO-treated control samples were calculated by using the comparative Ct method. Data are presented as means ± standard deviation (*n* = 3 per group). ^∗^ *P* < 0.05, ^∗∗∗^ *P* < 0.001.

**Figure 5:**
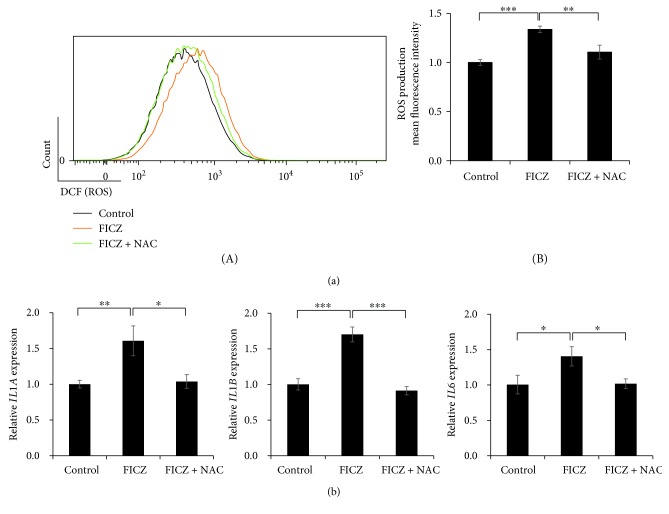
FICZ induces the expression of proinflammatory cytokine genes in a ROS- and AHR-dependent manner. (a) HaCaT cells were treated with DMSO (0.1%, control) or with FICZ (100 nM) in the presence or absence of NAC (5 mM) for 6 h, and ROS production was assessed by flow cytometry. Representative histograms of DCF fluorescence (A) are shown, along with the mean fluorescence intensities of DCF (ROS production) (B). Black: DMSO (0.1%, negative control); orange: FICZ (100 nM); green: FICZ (100 nM) plus NAC (5 mM). (b) HaCaT cells were treated with DMSO (0.1%, control) or FICZ (100 nM) in the presence or absence of NAC (5 mM) for 3 h. Expression of *IL1A*, *IL1B*, and *IL6* was measured by qRT-PCR. *ACTB* served as an internal control, and relative expression compared with those of DMSO-treated control samples was calculated by using the comparative Ct method. Data are presented as means ± standard deviation (*n* = 3 per group). ^∗^ *P* < 0.05, ^∗∗^ *P* < 0.01, ^∗∗∗^ *P* < 0.001.

**Figure 6:**
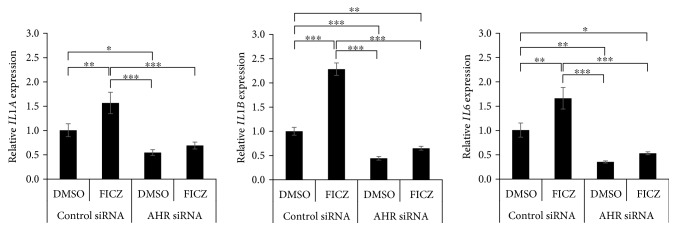
FICZ induces *IL6* expression in an AHR-dependent manner. HaCaT cells were transfected with negative control siRNA or AHR siRNA and further treated with DMSO (0.1%, control) or FICZ (100 nM) for 3 h. Expression of *IL1A*, *IL1B*, and *IL6* was measured by qRT-PCR. FICZ-induced elevation of *IL1A*, *IL1B*, and *IL6* mRNA expression was canceled in the AHR-knockdowned keratinocytes. Data are presented as means ± standard deviation (*n* = 3 per group). ^∗^ *P* < 0.05, ^∗∗^ *P* < 0.01, ^∗∗∗^ *P* < 0.001.

We next examined the effects of FICZ on proinflammatory cytokine expression in keratinocytes. FICZ upregulated the expression of *IL1A* (Figure 4(a)), *IL1B* (Figure 4(b)), and *IL6* (Figure 4(c)) but not of *TNF*, *IL8*, *IL36A*, *IL36B*, or *IL36G* (Supplementary Figure S4). To elucidate the relevance of ROS in the upregulation of *IL1A*, *IL1B*, and *IL6* expression, we treated keratinocytes with FICZ in the presence or absence of the antioxidant NAC. NAC potently canceled FICZ-induced ROS production (Figure 5(a)). In parallel, NAC abolished the FICZ-mediated upregulation of *IL1A*, *IL1B*, and *IL6* (Figure 5(b)). Because FICZ-induced ROS production was canceled in AHR-knockdowned keratinocytes (Figure 3), we further evaluated the effects of AHR knockdown on FICZ-induced proinflammatory cytokine expression. As expected, the FICZ-induced elevation of *IL1A*, *IL1B*, and *IL6* mRNA expression was canceled in AHR-knockdowned keratinocytes (Figure 6).

**Figure 7:**
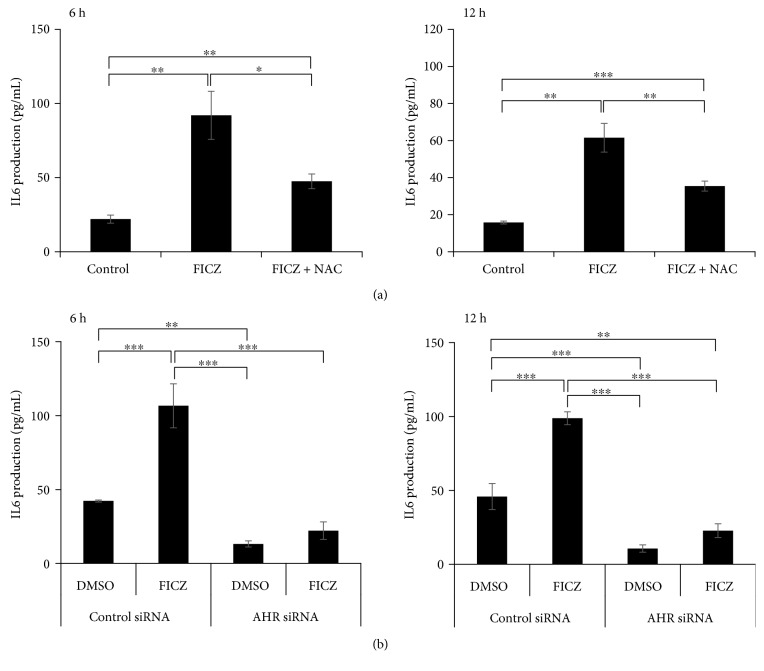
NAC and AHR siRNA inhibit FICZ-induced IL6 production. (a) HaCaT cells were treated with DMSO (0.1%, control) or FICZ (100 nM) in the presence or absence of NAC (5 mM) for 6 or 12 h. (b) HaCaT cells were transfected with negative control siRNA or AHR siRNA and then further treated with DMSO (0.1%, control) or FICZ (100 nM) for 6 or 12 h. Production of IL6 in the culture supernatant was measured by ELISA. Data are presented as means ± standard deviation (*n* = 3 per group). ^∗^ *P* < 0.05, ^∗∗^ *P* < 0.01, ^∗∗∗^ *P* < 0.001.

We next investigated the protein levels of IL1A, IL1B, and IL6 in culture supernatants. IL6 protein was released from keratinocytes and was present in the culture supernatant 6 and 12 h after incubation with FICZ, but this release was significantly inhibited in the simultaneous presence of NAC (Figure 7(a)). FICZ-induced upregulation of IL-6 production was also canceled in keratinocytes transfected with AHR siRNA after 6 and 12 h of FICZ treatment (Figure 7(b)). Although FICZ upregulated *IL1A* and *IL1B* mRNA expression, it did not induce the release of IL1A and IL1B, even 6, 12, 24, or 48 h after the incubation with FICZ (data not shown). As the IL6 production is dependent on NF-*κ*B activation in keratinocytes [5], we finally examined whether FICZ activates and induces the nuclear translocation of NF-*κ*B p65. As shown in Supplementary Figure S5, FICZ did induce the nuclear translocation of NF-*κ*B p65. These results indicated that FICZ upregulated the expression of *IL1A* and *IL1B* mRNAs as well as *IL6* mRNA expression and its protein production in a ROS- and AHR-dependent manner.

## 4. Discussion

It has been well documented by the assessment of CYP1A1 induction that UVB and its chromophore FICZ activate the AHR [6, 14, 15, 18]. The AHR is an authentic xenobiotic receptor for dioxins and is responsible for dioxin-mediated ROS generation and proinflammatory cytokine production [7–9]. Ligation of AHR induces the gene and protein expression of CYP1A1 [7–9]. The AHR-mediated ROS production is dependent on CYP1A1 since the ROS production is attenuated in CYP1A1-deficient cells and mice [9, 24]. However, it remains unknown whether FICZ is an active inducer of proinflammatory cytokine production and whether ROS contribute to the event in keratinocytes.

We demonstrated here that FICZ upregulated the CYP1A1 expression and that FICZ generated ROS in an AHR-dependent manner in HaCaT keratinocytes and upregulated the expression of *IL1A*, *IL1B*, and *IL6* in a ROS- and AHR-dependent manner. Production of IL6 was also upregulated by FICZ in a ROS- and AHR-dependent fashion. Previous studies have demonstrated that the UVB radiation upregulates *IL1B* and *IL6* expression [4, 25]. Similar to FICZ, our previous study found that UVB-induced IL-6 upregulation was dependent on AHR and ROS [4]. These findings stress the notion that FICZ is indeed an important chromophore that is partly responsible for the biophysical actions of UVB, including CYP1A1 induction, ROS generation, and proinflammatory cytokine production. However, UVB exposure activates not only AHR pathway but also multifaceted signal transduction pathways including caspase 1 and NF-*κ*B [3, 25]. In addition to AHR activation, the activation of caspase 1 is mandatory for the protein release of IL1B [25]. This fact may explain why the FICZ-AHR activation upregulated the mRNA expression of IL1B but not its protein release.

As HaCaT keratinocytes are immortalized keratinocytes, the major limitation of this study is whether FICZ induces similar biological response to normal human keratinocytes or not. In Supplementary Figure S6, using normal human keratinocytes, we confirmed that (1) FICZ upregulated *CYP1A1* expression as early as at 2 h after incubation, (2) FICZ-induced CYP 1A1 upregulation was canceled in the AHR-knockdowned keratinocytes, and (3) FICZ also induced a significant ROS production but at less extent compared to UVB exposure. Therefore, we assume that HaCaT and normal keratinocytes share similar biological behaviors against FICZ.

A recent study also revealed that FICZ acts as a potent UVA photosensitizer: the viability of keratinocytes is not affected by either FICZ or UVA alone; however, cotreatment with FICZ and UVA induces apoptosis of the majority of keratinocytes in association with the production of caspase 3 and heat shock protein 70 [22]. In addition, FICZ inhibits collagen production and accelerates its degradation by upregulating collagenase 1 (matrix metalloproteinase 1) production [26, 27]. These hazardous effects of FICZ overlap with the photoaging effects of UVB exposure. Although FICZ is a high-affinity agonist for the AHR [6], its biological importance is still controversial because of its low production after UVB exposure [28]. As FICZ is efficiently metabolized by CYP1A1 enzyme [15, 29, 30], rapid degradation of FICZ by CYP1A1 may be the reason for its low quantity.

## 5. Conclusions

The tryptophan-derived photoproduct FICZ is a potent AHR-ROS-proinflammatory cytokine inducer; the effects of which may partly responsible for the hazardous effects of UVB exposure.

## Figures and Tables

**Table 1:** Primer sequences for qRT-PCR.

Gene symbol		Sequence
IL1A	Sense	5′- AGATGCCTGAGATACCCAAAACC -3′
	Antisense	5′- CCAAGCACACCCAGTAGTCT -3′

IL1B	Sense	5′- ATGATGGCTTATTACAGTGGCAA -3′
	Antisense	5′- GTCGGAGATTCGTAGCTGGA -3′

IL6	Sense	5′- ACTCACCTCTTCAGAACGAATTG -3′
	Antisense	5′- CCATCTTTGGAAGGTTCAGGTTG -3′

IL8	Sense	5′- CTGGCCGTGGCTCTCTTG -3′
	Antisense	5′- CCTTGGCAAAACTGCACCTT -3′

TNF	Sense	5′- GAGGCCAAGCCCTGGTATG -3′
	Antisense	5′- CGGGCCGATTGATCTCAGC -3′

IL36A	Sense	5′- TGGGTTCTTCAGGACCAGAC -3′
	Antisense	5′- GATGGGGTTCCCTCTGTCTT -3′

IL36B	Sense	5′- TTCAGGGCAAGCCTACTTTG -3′
	Antisense	5′- TTCCCATGAAGCAGCTCTCT -3′

IL36G	Sense	5′- GAAACCCTTCCTTTTCTACCGTG -3′
	Antisense	5′- GCTGGTCTCTCTTGGAGGAG -3′

ACTB	Sense	5′- ATTGCCGACAGGATGCAGA -3′
	Antisense	5′- GAGTACTTGCGCTCAGGAGGA -3′

## Data Availability

The gene expression data, protein production data, and ROS production data used to support the findings of this study are included within the article and supplementary materials.

## Supplementary Materials

Supplementary Materials Supplementary Figure S1: FICZ does not affect the viability of HaCaT keratinocytes. The viability of HaCaT cells treated with DMSO (0.1%, control) or FICZ (1, 10, or 100 nM or 1 or 10 *μ*M) for 6 or 24 h was assessed by using a WST-8 formazan-based method. Data are presented as means ± standard deviation (*n* = 5 per group). Supplementary Figure S2: FICZ induces cytoplasmic to nuclear translocation of AHR. HaCaT cells were treated with DMSO (0.1%, control) or FICZ (1 and 100 nM) for 6 h and were immunostained with AHR. AHR was mainly localized in the cytoplasm in DMSO-treated control, while its nuclear translocation was induced by FICZ even at a concentration of 1 nM (arrows). Supplementary Figure S3: FICZ induces intracellular glutathione (GSH) reduction. HaCaT cells were treated with DMSO (0.1%, control) or FICZ (1, 10, 100, or 1000 nM) for 6 h and analyzed by GSH reduction assay. FICZ (10 to 1000 nM) did reduce the intracellular level of glutathione, implicating the production of ROS. Data are presented as means ± standard deviation (*n* = 3 per group). Supplementary Figure S4: expression of proinflammatory cytokine mRNAs in keratinocytes treated with FICZ (100 nM). Expression of IL8, TNF, IL36A, IL36B, and IL36G in the same sample as in Figure 4 was measured by qRT-PCR. Data are presented as means ± standard deviation (*n* = 3 per group). Supplementary Figure S5: FICZ activates NF-*κ*B signaling. HaCaT cells were treated with DMSO (0.1%, control) or FICZ (100 nM), and nuclear translocation of NF-*κ*B p65 was evaluated by western blot. Representative images of the bands (left panel) and density of nuclear NF-*κ*B p65 relative to DMSO-treated control (right panel) are shown. Nuclear lamin B1 protein was used as the internal control. Data are presented as means ± standard deviation (*n* = 3 per group). Supplementary Figure S6: FICZ induces CYP1A1 and ROS production in normal human keratinocytes. Normal human epidermal keratinocytes (NHEKs) were (a) treated with DMSO (0.1%, control) or FICZ (100 nM) for 2, 4, and 6 h or were (b) transfected with control siRNA or AHR siRNA, further treated with DMSO (0.1%) or FICZ (100 nM), and assessed for the expression of CYP1A1 mRNA. (c) NHEKs were irradiated with UVB (30 mJ/cm2) or treated with FICZ (100 nM), and ROS production at 6 h posttreatment was evaluated by flow cytometry. Representative image of histogram (left panel) and mean fluorescence intensity of DCF (right panel) are shown. Data are presented as means ± standard deviation (*n* = 3 per group).
