# Supplementary material for: Tryptophan Photoproduct FICZ Upregulates IL1A, IL1B, and IL6 Expression via Oxidative Stress in Keratinocytes
Source: Oxid Med Cell Longev. 2018 Nov 25;2018:9298052. doi: 10.1155/2018/9298052 (PMC6286782; doi:10.1155/2018/9298052)
Supplement: Supplementary Materials — Supplementary Figure S1: FICZ does not affect the viability of HaCaT keratinocytes. The viability of HaCaT cells treated with DMSO (0.1%, control) or FICZ (1, 10, or 100 nM or 1 or 10 μM) for 6 or 24 h was assessed by using a WST-8 formazan-based method. Data are presented as means ± standard deviation (n = 5 per group). Supplementary Figure S2: FICZ induces cytoplasmic to nuclear translocation of AHR. HaCaT cells were treated with DMSO (0.1%, control) or FICZ (1 and 100 nM) for 6 h and were immunostained with AHR. AHR was mainly localized in the cytoplasm in DMSO-treated control, while its nuclear translocation was induced by FICZ even at a concentration of 1 nM (arrows). Supplementary Figure S3: FICZ induces intracellular glutathione (GSH) reduction. HaCaT cells were treated with DMSO (0.1%, control) or FICZ (1, 10, 100, or 1000 nM) for 6 h and analyzed by GSH reduction assay. FICZ (10 to 1000 nM) did reduce the intracellular level of glutathione, implicating the production of ROS. Data are presented as means ± standard deviation (n = 3 per group). Supplementary Figure S4: expression of proinflammatory cytokine mRNAs in keratinocytes treated with FICZ (100 nM). Expression of IL8, TNF, IL36A, IL36B, and IL36G in the same sample as in Figure 4 was measured by qRT-PCR. Data are presented as means ± standard deviation (n = 3 per group). Supplementary Figure S5: FICZ activates NF-κB signaling. HaCaT cells were treated with DMSO (0.1%, control) or FICZ (100 nM), and nuclear translocation of NF-κB p65 was evaluated by western blot. Representative images of the bands (left panel) and density of nuclear NF-κB p65 relative to DMSO-treated control (right panel) are shown. Nuclear lamin B1 protein was used as the internal control. Data are presented as means ± standard deviation (n = 3 per group). Supplementary Figure S6: FICZ induces CYP1A1 and ROS production in normal human keratinocytes. Normal human epidermal keratinocytes (NHEKs) were (a) treated with DMSO (0.1%, control [file 9298052.f1.pdf]

Supplementary Figure S1

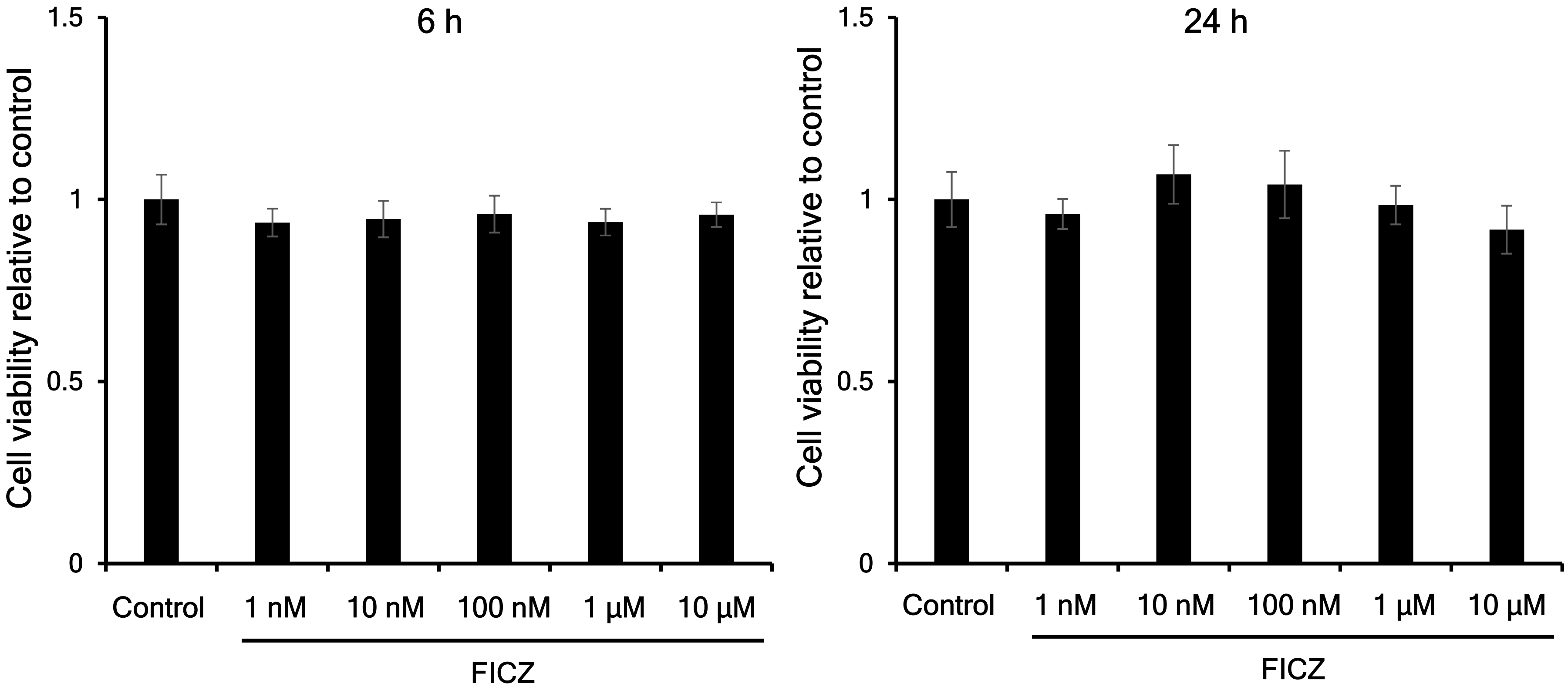

Supplementary Figure S1: FICZ does not affect the viability of HaCaT keratinocytes.
The viability of HaCaT cells treated with DMSO (0.1%, control) or FICZ (1, 10, or 100 nM or 1 or 10 µM) for 6 or 24 h was assessed by using a WST-8 formazan-based method. Data are presented as means ± standard deviation (n = 5 per group).

Supplementary Figure S2

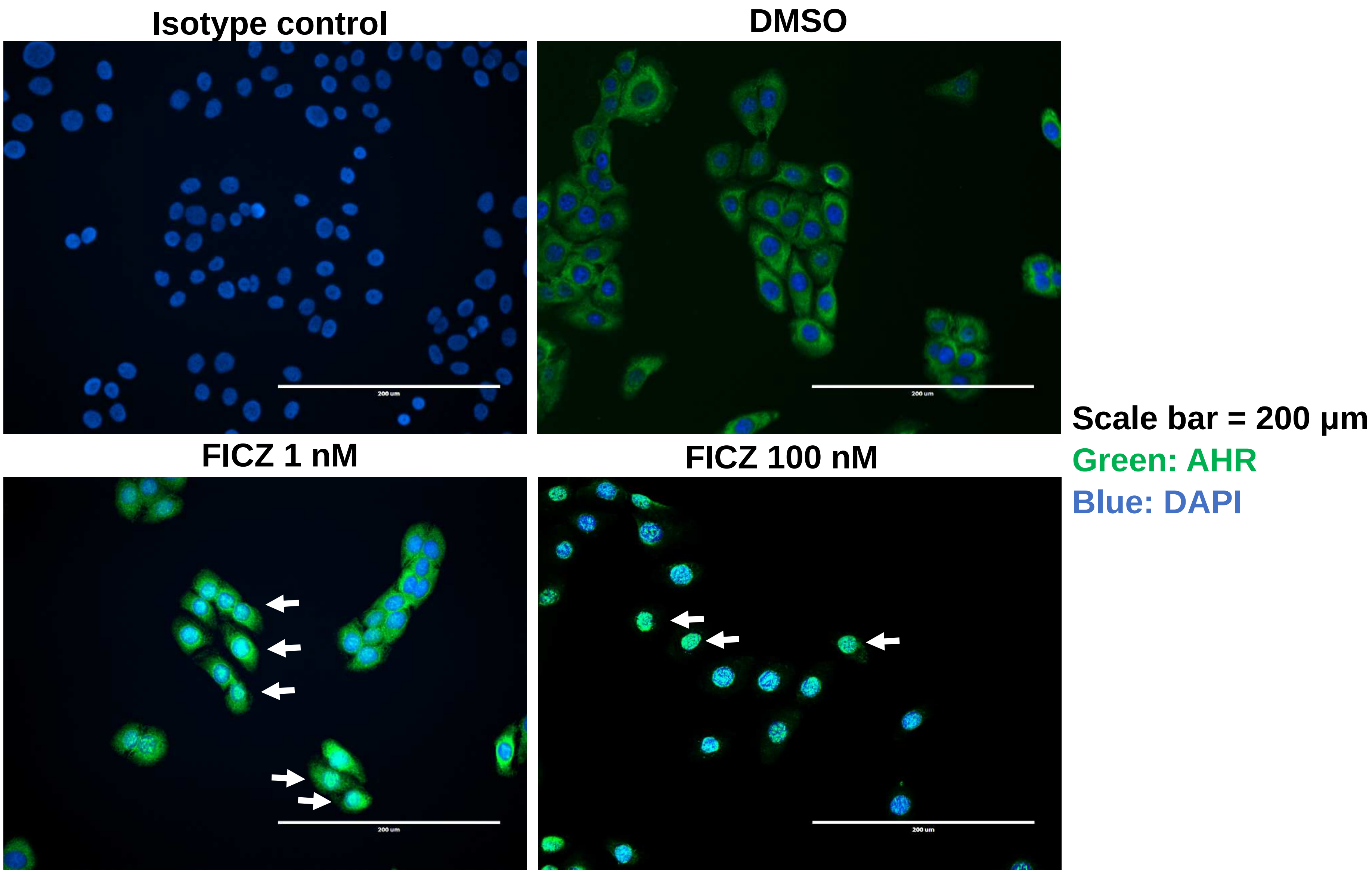

Supplementary Figure S2: FICZ induces cytoplasmic to nuclear translocation of AHR.
HaCaT cells were treated with DMSO (0.1%, control) or FICZ (1 and 100 nM) for 6 h and were immunostained with AHR. AHR was mainly localized in the cytoplasm in DMSO-treated control, while its nuclear translocation was induced by FICZ even at a concentration of 1 nM (arrows).

Supplementary Figure S3

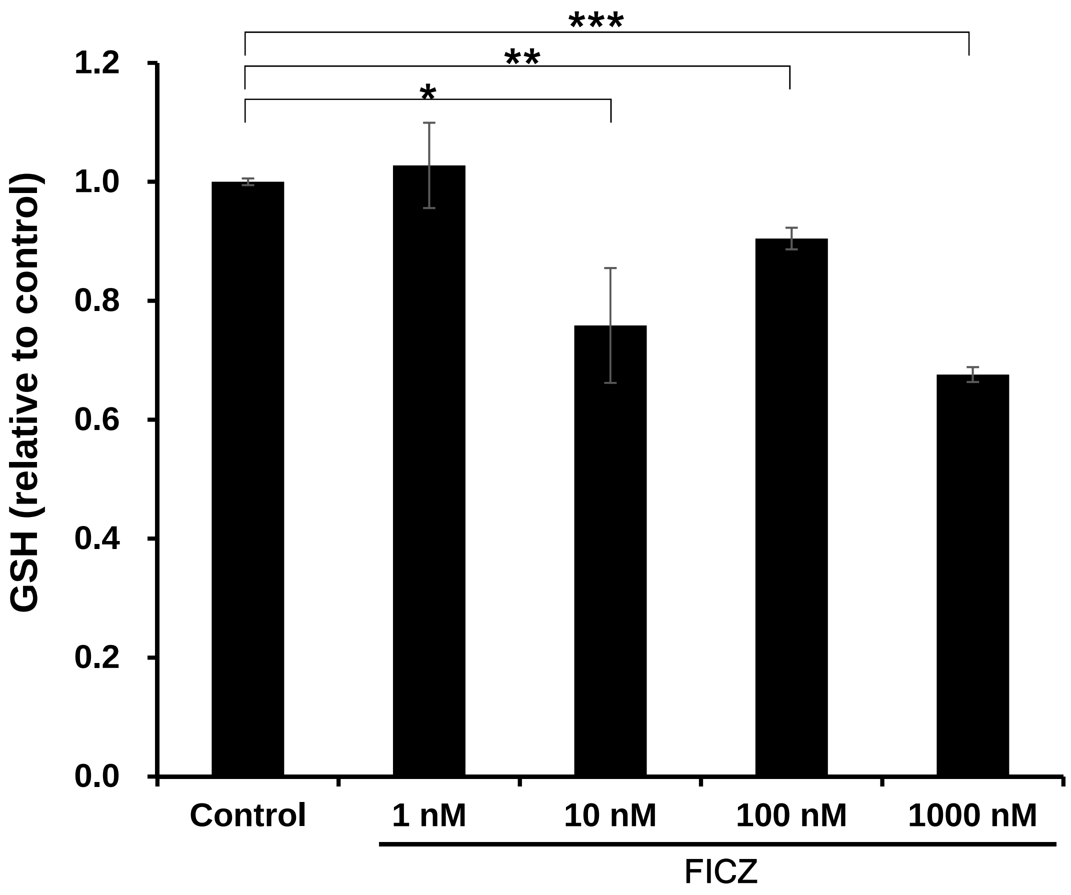

Supplementary Figure S3: FICZ induces intracellular glutathione (GSH) reduction.
HaCaT cells were treated with DMSO (0.1%, control) or FICZ (1, 10, 100 or 1000 nM) for 6 h and analyzed by GSH reduction assay. FICZ (10 to 1000 nM) did reduce the intracellular level of glutathione, implicating the production of ROS. Data are presented as means ± standard deviation (n = 3 per group).

Supplementary Figure S4

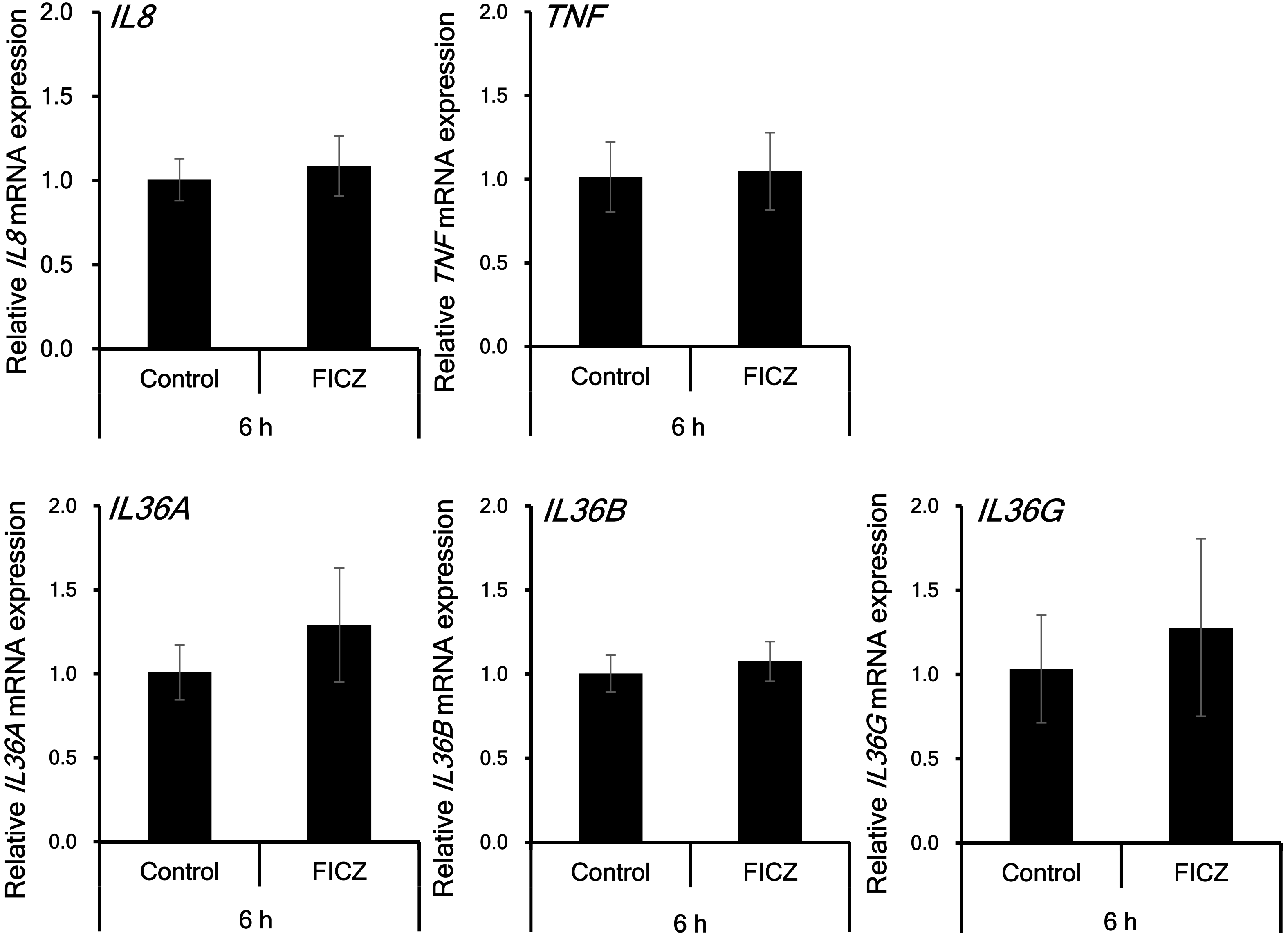

Supplementary Figure S4: Expression of proinflammatory cytokine mRNAs in keratinocytes treated with FICZ (100 nM).

Expression of *IL8*, *TNF*, *IL36A*, *IL36B*, and *IL36G* in the same sample as in Figure 4 was measured by qRT-PCR. Data are presented as means ± standard deviation (n = 3 per group).

Supplementary Figure S5

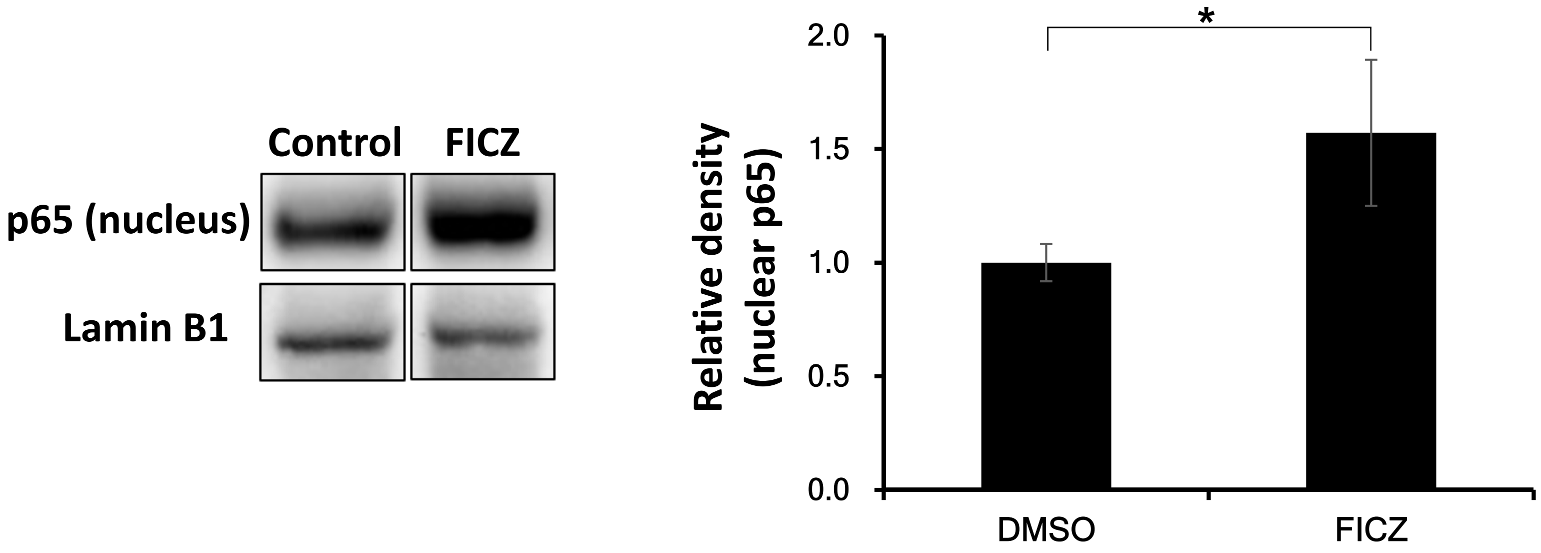

Supplementary Figure S4: FICZ activates NF-κB signaling.
HaCaT cells were treated with DMSO (0.1%, control) or FICZ (100 nM) and nuclear translocation of NFκB p65 was evaluated by western blot. Representative image of bands (left panel) and density of nuclear NF-κB p65 relative to DMSO-treated control (right panel) are shown. Nuclear Lamin B1 protein was used as internal control. Data are presented as means ± standard deviation (n = 3 per group).

Supplementary Figure S6

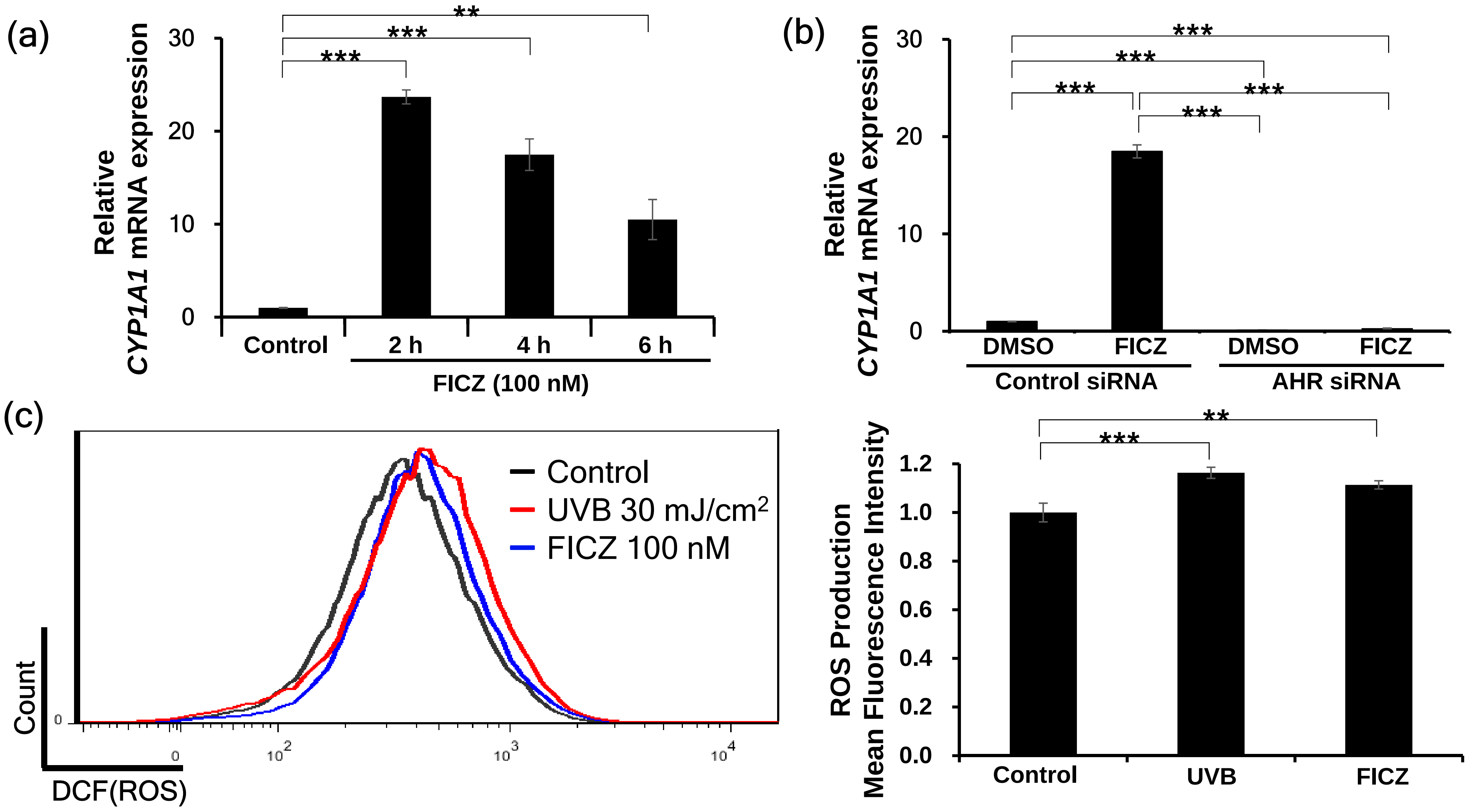

Supplementary Figure S6: FICZ induces CYP1A1 and ROS production in normal human keratinocytes.
Normal human epidermal keratinocytes (NHEKs) were (a) treated with DMSO (0.1%, control) or FICZ (100 nM) for 2, 4 and 6 h, or were (b) transfected with control siRNA or AHR siRNA, further treated with DMSO (0.1%) or FICZ (100 nM), and assessed for the expression of *CYP1A1* mRNA. (c) NHEKs were irradiated with UVB (30 mJ/cm^2) or treated with FICZ (100 nM) and ROS production at 6 h-post treatment was evaluated by flow cytometry. Representative image of histogram (left panel) and mean fluorescence intensity of DCF (right panel) are shown. Data are presented as means ± standard deviation (n = 3 per group).
